# Magnetic resonance elastography (MRE) for the evaluation of fibrosis in patients with benign uterine disorders: a systematic review

**DOI:** 10.1007/s00261-026-05374-8

**Published:** 2026-02-05

**Authors:** Sabrine Q. Kol, Nienke P.M. Wassenaar, Robert A. de Leeuw, Shandra Bipat

**Affiliations:** 1 Department of Radiology and Nuclear Medicine, Amsterdam University Medical Centers, Amsterdam, Netherlands; 2 Imaging and Biomarkers, Cancer Center Amsterdam, Amsterdam, Netherlands; 3 Department of Obstetrics and Gynaecology, Amsterdam University Medical Centers, Amsterdam, Netherlands; 4 Amsterdam Reproduction and Development (AR&D), Research Institute, Amsterdam, Netherlands

**Keywords:** Magnetic resonance imaging, Magnetic resonance elastography, Uterine leiomyomas, Adenomyosis, Benign uterine pathology, Systematic review

## Abstract

**Objective:**

To review magnetic resonance elastography (MRE) techniques and evaluate the feasibility of quantifying the extent of fibrosis in patients with benign uterine disorders.

**Materials and methods:**

A systematic search of the MEDLINE and EMBASE databases was performed for identifying relevant articles published between January 1, 2007, and September 2025. Studies meeting predefined inclusion criteria were selected. Two independent reviewers extracted data on study design, patient population, MRI protocol characteristics, MRE parameters and MRI/MRE features. Mean stiffness (kPa), including standard deviation were either extracted or calculated. The same reviewers also assessed the methodological quality of each study.

**Results:**

Six studies comprising a total of 162 patients (mean age range: 40.5 to 49 years) were included. Five studies focused on stiffness measurements in leiomyomas, while one study investigated the feasibility of stiffness measurements in patients with adenomyosis. All studies were classified as either pilot or feasibility studies. In studies reporting reproductive status, most patients were premenopausal (89 out of 125). The mean stiffness in evaluating leiomyomas ranged from 3.02 to 7.10 kPa across the included studies, resulting in a pooled mean stiffness of 4.72 ± 1.83 kPa.

Uterine stiffness was higher in women with adenomyosis (2.93 kPa; range, 2.34–3.39 kPa) than in the healthy volunteer (2.04 kPa). Two studies correlated stiffness measurements with histopathological findings of fibrosis. All included studies were rated as having good methodological quality.

**Conclusions:**

Despite the small number of studies, current findings suggest that MRE is a feasible imaging modality for measuring fibrosis.

**Supplementary Information:**

The online version contains supplementary material available at 10.1007/s00261-026-05374-8.

## Introduction

Benign uterine disorders encompass a heterogeneous group of gynaecological pathologies, including leiomyomas (fibroids) and adenomyosis. Leiomyomas represent the most prevalent uterine mass, reported to occur in over 70% of women by the onset of menopause [1]. Adenomyosis, characterized by the presence of endometrial glands and stroma within the myometrium, has an estimated prevalence ranging between 5 and 70%, depending on the diagnostic method applied [2], and is commonly associated with deep endometriosis (DE) [3]. Collectively, these disorders represent a significant clinical burden, not only because of their impact on quality of life but also due to their implications for fertility and reproductive health [4–6].

Although these benign uterine disorders differ in their pathogenesis and clinical presentation, they all are associated with alterations in the extracellular matrix and increasing amounts of fibrosis. Leiomyomas are defined by smooth muscle proliferation within a fibrotic stroma [7], while adenomyosis results from the pathological invasion of endometrial tissue into the myometrium, which triggers smooth muscle hyperplasia and hypertrophy, ultimately leading to fibrotic remodeling [8, 9]. This fibrotic remodeling is driven by fibroblast activity and excessive extracellular matrix deposition, which play a critical role in symptomatology, including pain and abnormal uterine bleeding [10]. Moreover, fibrotic changes can impair uterine contractility and increase the risk of obstetric complications, including uterine rupture, which has been reported to be more frequent in adenomyosis than in other uterine pathologies [11, 12]. Adenomyosis may develop from the endometrium outward or from the serosal surface inward, the latter form being strongly associated with deep endometriosis, which undergoes extensive fibrotic transformation contributing to its infiltrative behavior [13]. In leiomyomas, fibrosis not only contributes to lesion growth and symptom severity but also impacts treatment response, influencing outcomes of interventions such as uterine artery embolization [14]. Across both conditions, the degree of fibrosis adds complexity to medical and surgical management, highlighting the importance of incorporating fibrosis assessment into pre-treatment planning [15, 16]. Consequently, non-invasive imaging techniques capable of evaluating tissue stiffness and detecting fibrosis in vivo, such as elastography, may offer valuable diagnostic and prognostic insights across the spectrum of benign uterine pathologies.

Elastography is an imaging technique used to assess tissue stiffness and can be performed with either ultrasound or magnetic resonance imaging (MRI). Ultrasound elastography may be carried out using strain elastography, which is qualitative and operator-dependent, or shear wave elastography, which provides quantitative values but has technical limitations [17]. Both techniques face reproducibility challenges, particularly due to the selection of the region of interest (ROI) in heterogeneous lesions. In benign gynaecology, ultrasound elastography (USE) has been applied to the evaluation of the normal uterus, the myometrium (leiomyomas and adenomyosis), the endometrium (polyps), and pelvic endometriosis [18–22]. Acar et al. [19] demonstrated that myometrial stiffness measured with shear wave elastography (SWE) was significantly higher in adenomyosis than in healthy controls. Similarly, Vora et al. [20] reported increased stiffness in submucosal leiomyomas and focal adenomyomas.

Magnetic resonance elastography (MRE) also provides quantitative measurements of tissue stiffness, achieved by applying low-frequency vibrations and tracking shear wave propagation with MRI. MRE is already used in clinical practice for liver fibrosis [23] and is being explored in areas such as pancreatic [24], cardiovascular [25], and neurological diseases [26]. The limitations of MRE include its high cost, limited availability, longer examination time, MRI-related contraindications, and the need for patients to remain still during image acquisition. Nevertheless, MRE offers several advantages over USE, including being able to assess whole organs (e.g. the uterus), not being as limited by depth or acoustic windows (e.g. body habitus, ascites), providing quantitative measurements and not being operator dependent [27,28]. Furthermore, because MRE is performed within the context of an MRI examination, it also delivers high-resolution anatomical information along with tissue stiffness data.

This study aims to systematically review MRE techniques and reported outcomes related to the use of MRE in quantifying fibrosis in patients with benign uterine disorders.

## Materials and methods

### Study design

This systematic review was conducted and reported in accordance with the Preferred Reporting Items for Systematic Reviews and Meta-analyses (PRISMA) guidelines: as outlined by Page et al. [29].

### Search strategy

A literature search was performed in the MEDLINE and EMBASE databases to identify relevant articles published from 2007 to date of search (Sept 1, 2025), as MRE was first introduced as a clinical test in 2007. The search strategy included the following search terms: “Magnetic Resonance Imaging” AND “Elastography” AND “Fibroids” OR “Leiomyomas” OR “Endometriosis” OR “Adenomyosis”. The search strategy is described in detail in Supplement A. To identify additional articles, the citation indexes and the reference lists of relevant articles were checked.

### Selection of relevant articles

The title and/or abstract of all retrieved articles were screened for potential relevance by Reviewer 2 (X1), a methodologist with extensive experience in systematic reviews. The following were excluded from further analysis: duplicates, conference abstracts, clinical registry entries, editorials, commentaries, letters-to-the-editor, non-relevant literature (e.g., not disease-related or involving other imaging techniques), narrative reviews, and case-reports.

### Inclusion criteria

Subsequently, the full texts of the remaining articles were assessed by the same reviewer. The inclusion criteria were: 1) studies involving patients with a benign uterine disorder (including but not limited to: leiomyomas, deep endometriosis and/or adenomyosis); 2) evaluation of fibrosis using MRE. All articles meeting these criteria were included for further data extraction.

### Data-extraction

Data-extraction was performed independently by two reviewers, X1 and X2, the latter being an abdominal radiologist with five years of dedicated experience in pelvic MRI. Any discrepancies between the reviewers were resolved through discussion. The following data were extracted: 1) Study characteristics, 2) Study population characteristics, 3) MRI protocol characteristics, 4) MRE parameters and main outcomes, 5) MRI and MRE features of leiomyomas, 6) Methodological quality of the included studies.

### Study characteristics

The following data were extracted: 1) First author and year of publication, 2) Study period, 3) Country of origin, 4) Setting (academic or other), 5) Study type (pilot, feasibility or cohort), 6) Study design (single-center or multi-center, multi-center indicating involvement of authors from different institutions), 7) Department of first author, 8) Data collection method (retrospective or prospective), 9) Funding information, 10) Ethical approval status (No, Yes – whether informed consent was waived or obtained), and 11) Disclosure of conflict of interest.

### Study population characteristics

The following study population-related data were obtained: 1) Inclusion and exclusion criteria, 2) Number of included patients, 3) Age (reported as mean ± standard deviation (SD) and/or range), 4) Body Mass Index (BMI) (reported as mean ± SD or median and range), 5) Menopausal status (premenopausal, perimenopausal or postmenopausal), 6) Symptoms, 7) Ethnicity, and 8) Type of surgery performed.

### MRI protocol characteristics

The following technical aspects of MRI were extracted: 1) MRI vendor, 2) Magnetic field strength, 3) Type of coil used (body, phased array, or other), 4) MRI sequences performed (e.g., fat-saturated T1, post-contrast T1, T2-weighted, T2 HASTE, diffusion-weighted imaging (DWI) and/or dynamic contrast-enhanced (DCE) imaging).

### MRE parameters

The summarized technical aspects of MRE included: 1) Mechanical wave frequency (in Hertz), 2) Method used to define the region of interest (ROI), 3) MRE post processing method, 4) Stiffness measurements reported for benign uterine disorder.

### MRI and MRE features of leiomyomas

The following data were extracted: 1) Leiomyoma volume (cm^3^), 2) Leiomyomadiameter (cm), 3) Number of leiomyomas, 4) Location of leiomyoma in uterus wall, 5)Leiomyoma characteristics on T2-WI, 6) Leiomyoma characteristics on post-contrast imaging and, 7) MRE data of stiffness of leiomyoma in kPa (mean ± SD and/or median with ranges). In case MRE data were presented for any subgroups, these data were also extracted.

### Statistical analysis

Due to the expected small number of studies and limited patient cohorts, mostly originating from pilot and feasibility investigations, a formal meta-analysis was not conducted. Instead, pooled means and pooled standard deviations were estimated by weighting each study according to its sample size, mean, and standard deviation, thereby giving greater influence on studies with larger population. Details on pooling of the means and the standard deviations are given in Supplement B.

### Methodological quality assessment

The methodological quality of the included studies was evaluated based using the National Institutes of Health (NIH) quality Assessment Tool for Case Series Studies (https://www.nhlbi.nih.gov/health-topics/study-quality-assessment-tools) (Supplement C). As all articles described cross-sectional studies (no cohort with exposure, no diagnosis with reference standard and no treatment), we choose a checklist for cross-sectional studies to assess the methodological quality. And as these cross-sectional studies were predominantly pilot or feasibility studies, we choose the NIH checklist for case series (low number of patients in each study) [30].

This tool consists of several questions addressing key domains of bias including, selection bias, performance bias, detection bias and attrition bias (i.e., lost to follow-up). Each question was rated with “yes”, “no”, “other” (“cannot determine” (CD), “not applicable” (NA), or “not reported” (NR)). Finally, overall quality was determined based on the outcomes of the questions. If 6–8 questions were answered with YES, the quality rating of the study was assessed as “Good”. If 4–5 questions were answered with YES, the quality of the study was assessed as “Fair’’. In all of the other cases the quality was assessed as “Poor”.

## Results

### Search strategy, selection and inclusion

**Fig. 1:**
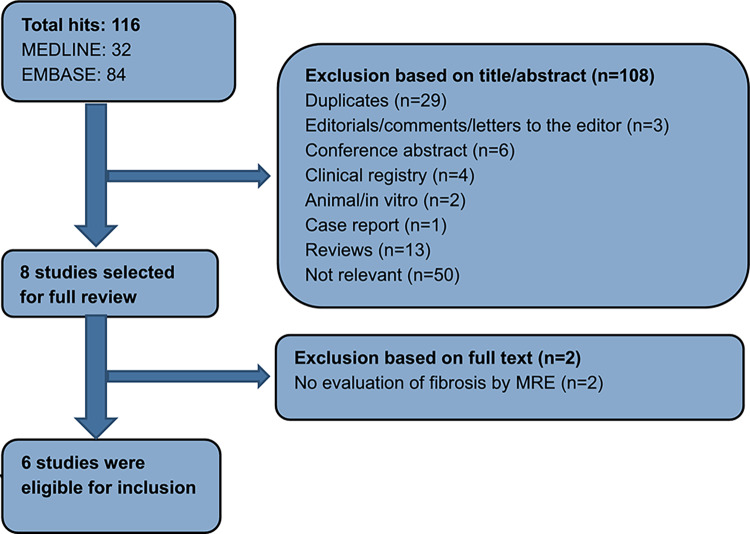
Search, selection and inclusion of relevant articles

The initial search of the MEDLINE and EMBASE databases yielded 116 records (Fig. 1). After screening titles and abstracts, 108 studies were excluded for not meeting the inclusion criteria. The full texts of the remaining eight articles were reviewed in detail, resulting in the exclusion of two additional studies. Ultimately six articles were included for data extraction, five evaluating MRE in patients with leiomyomas [31–35] and one evaluating MRE in patients with adenomyosis [36].

### Study characteristics

All included studies were conducted in academic settings and were classified as either feasibility (2 studies) or pilot studies (4 studies). Five out of six studies were prospectively performed and all had received ethical approval. Furthermore, 5 of the 6 studies focussed on stiffness measurements in leiomyomas, while one study investigated the feasibility of stiffness measurements in patients with adenomyosis. Additional study details are summarized in Table 1.

**Table 1:** Study characteristics of included articles

Leiomyoma										
Author	Department of first authors	Country of origin	Study type	Study design	Study Setting	Study period	Data collection	Information on funding	Information on ethical approval	Conflict of interest
Stewart^@^, [ 31]	Obstetrics Gynecology	U.S.A	Feasibility	Single-center	Academic	Apr. 2008-Mar. 2009	Prospective	Yes, with funding disclosed	Approved and informed consent obtained	None declared
Jondal^@^, [32]	Radiology	U.S.A	Pilot	Single-center	Academic	N. A.*	Prospective	Yes, with funding disclosed	Approved and informed consent obtained	Yes, declared
Ichikawa, [33]	Radiology	Japan	Pilot	Multi-center^**#**^	Academic	Feb. 2013- Dec. 2014	Retrospective	Not reported	Approved and informed consent obtained	None declared
Obrzut, [34]	Biophysics	Poland	Pilot	Multi-center^**#**^	Academic	Sept. 2016- Feb. 2017	Prospective	Yes, with funding disclosed	Approved and informed consent obtained	Not reported
Aphinives, [35]	Radiology	Thailand	Pilot	Single-center	Academic	Sept. 2020- Oct. 2021	Prospective	Yes, with funding disclosed	Approved and informed consent obtained	None declared
Adenomyosis										
Author	Department of first authors	Country of origin	Study type	Study design	Study Setting	Study period	Data collection	Information on funding	Information on ethical approval	Conflict of interest
Jain, [36﻿]	Centre for Reproductive Health	United Kingdom	Feasibility	Multi-center^**#**^	Academic	N. A.*	Prospective	Yes, with funding disclosed	Approved and informed consent obtained	Not reported

^*^N.A. Not available, ^**#**^Multi-center indicating involvement of authors from different institutions; ^@^ studies performed at the same institute

### Study population characteristics

A total of 162 patients were included across all studies: 157 patients underwent MRE to assess fibrosis in uterine leiomyomas, 4 underwent MRE for the evaluation of fibrosis in adenomyosis, and one was a healthy volunteer. The reported mean age ranged from 40.5 to 49 years. In the studies where reproductive status was documented, most patients were premenopausal (89 out of 125). Other patient characteristics are listed in Table 2.

**Table 2:** Study population characteristics of included articles

Leiomyoma								
Author	Inclusion/ exclusion criteria	No. of patients	Age (years)	BMI (kg/m2)	Reproductive status (Pre-/peri-/post-menopausal)	Symptoms	Ethnicity	Treatment (# of patients)
Stewart, [31]	Planned for surgical excision of uterine leiomyomas	6	Mean: 42 ± 10 Range: 34–60	Mean: 28.9 ± 6.2 Range: 23–38	Pre: 5 Post: 1	Enlarged uterus/fibroid: 3 Menorrhagia: 11 Bulk symptoms: 1 Degenerating fibroid & preterm labor: 1	Caucasian: 3 Asian: 2 African-American: 1	Hysterectomy: 3 Myomectomy: 2 Diagnostic hysteroscopy: 1
Jondal, [32]	Patients between 18 and 89 years scheduled for a pelvic MRI for uterine fibroids or other uterine problems®	102	Mean: 44 ± 8.8	N.R	Pre: 68 Peri: 29 Post: 5	Menorrhagia: 89 Dysmenorrhea: 37 Increased urinary frequency: 51 Pain or pressure in abdomen/back: 65	Caucasian: 82 African-American: 11 Other: 9	None: 39 FUS: 13 UAE: 15 Surgery: 35
Ichikawa, [33]	Patients who underwent MRgFUS for uterine fibroids	11	Mean: 45.5 ± 4.4 range 38–52	N.R	N.R	Hypermenorrhoea: 8 Abdominal tightness: 2 Increased urinary frequency: 1	N.R	MRgFUS: 26*
Obrzut, [34]	Patients with symptomatic leiomyomas, who underwent surgical treatment	12	Mean 40.5 range 26–61	Median 22.45 range 19.16–27.24	Pre: 11 Post: 1	N.R	N.R	Hysterectomy: 7 Myomectomy: 5
Aphinives, [35]	> 18 years old, diagnosed with myoma uteri, and requested for pelvic MRI Excluded: patients with pregnancy or emergency medical conditions	26	Mean: 49 range 26–70	N.R	N.R	N.R	N.R	N.A

Adenomyosis								
Author	Inclusion/ exclusion criteria	Number of patients	Age (years)	BMI (kg/m2)	Reproductive status (Pre-/peri-/post-menopausal)	Symptoms	Ethnicity	Treatment (# of patients)
Jain, [36]	Patients with suspected adenomyosis and heavy menstrual bleeding diagnosed by TVUS	5^α^	N.R	N.R	Pre: 5	Heavy menstrual bleeding: 4 None: 1^α^	N.R	Hysterectomy: 2

*N.R.: not reported, N.A.: not applicable, FUS: Focused Ultrasound Surgery, UAE: Uterine artery embolization, TVUS: transvaginal ultrasound, MRgFUS: Magnetic Resonance-guided Focused Ultrasound Surgery*

®: all patients included had uterine leiomyomas, *: refers to number of leiomyomas treated not number of patients

^α^: including 1 healthy volunteer

### MRI protocol characteristics & MRE parameters

Four out of the six studies used a 1.5-T MRI, and 2 studies used a 3-T MRI. All patients were placed in supine position in the MRI scanner for image acquisition. None of the studies reported the use of spasmolytics or other preparation protocols as part of their imaging procedures. MRE was performed using phased-array coils to ensure adequate signal reception across the pelvic region. A passive driver was placed on the lower abdomen, directly over the uterus, and secured with a belt in three studies [33, 35, 36]. Aphinives et al. [35] reported using a soft pad placed beneath the passive driver to decrease patient vibrating sensations. The passive driver was connected via a flexible tube to an active driver, which was located outside the MRI suite, typically in the equipment room. The active drivers generated mechanical vibrations all at the frequency of 60 Hz, which was transmitted through the tube to the passive driver and subsequently propagated through the abdominal wall to the uterus.

Shear wave imaging was evaluated in three studies [31, 32, 34] using a modified two-dimensional (2D) gradient-recalled echo–based elastography pulse sequence. In a subset of patients in the Jondal et al. [32] study, additional three-dimensional (3D) multi-slice spin-echo–based planar imaging sequences were acquired, while Jain et al. [36] employed a 3D EPI MRE approach.

Following image acquisition, ROIs were selected. In three studies, the person responsible for delineating the ROIs was specified: in two studies ([31, 33], a radiologist performed this task, while in Aphinives et al. [35], an MRI technician was responsible. Additionally, three studies reported using the T2-weighted images as a guide when defining the ROIs, to avoid areas of degeneration in the leiomyoma, identified by T2 hyperintense areas.

Stiffness values were reported across studies using a combination of mean, median, standard deviation, and, in some cases, minimum and maximum values (kPa). Stewart et al.[31] additionally presented histograms of stiffness distributions.

None of the studies reported any adverse events during the MRE examination.

The MRI protocol characteristics and MRE technical features are summarized in Supplements D and E respectively. Additional MRE parameters and outcomes are listed in Table 3**.**

**Table 3:** MRE Parameters and Main Outcomes

Author	Wave Frequency (Hz)	Region of Interest (ROI) Placement	Stiffness Measurements Reported (kPa)	Main Outcome of Study
Stewart, [31]	60	ROIs were manually drawn by radiologists within the uterine leiomyoma using the stiffness maps	Mean stiffness, SD and histograms of tissue stiffness	Assess the in vivo stiffness of uterine leiomyomas using MRE
Jondal, [32]	60	ROIs were manually drawn on the largest leiomyoma, guided by corresponding T2-weighted images	Mean stiffness and SD of the ROIs	Correlate fibroid MRE stiffness with MRI characteristics
Ichikawa, [33]	60	Two radiologists placed ROIs within the uterine fibroid on stiffness maps, guided by T2-weighted images, avoiding areas of degeneration	Mean stiffness pre-and posttreatment	Evaluate usefulness of MRE for predicting treatment outcomes of patients receiving MRgFUS*
Obrzut, [34]	60	ROIs were manually drawn on the largest leiomyoma, guided by corresponding T2-weighted images	Mean stiffness and SD were reported	Investigated stiffness of leiomyomas in correlation with histopathologic composition
Aphinives, [35]	60	ROIs for the whole uterus were drawn manually on axial FFE images by an MRI technologist	Average, median, minimum, maximum stiffness and SD	Assess the feasibility of MRE in evaluating uterine fibroid stiffness in Thai patients
Jain, [36]	60	ROI demarcating the whole uterus as appeared on T2-WI and transferred and superimposed on the relevant stiffness map	Global estimated uterine stiffness	Asses the feasibility to measure uterine stiffness in adenomyosis and a healthy volunteer, and correlate findings with histology in 2 cases

^*^MRgFUS: MRI Guided Focused Ultrasound, FFE: fast field echo

### Leiomyomas

#### MRI and MRE features of leiomyomas

**Fig. 2:**
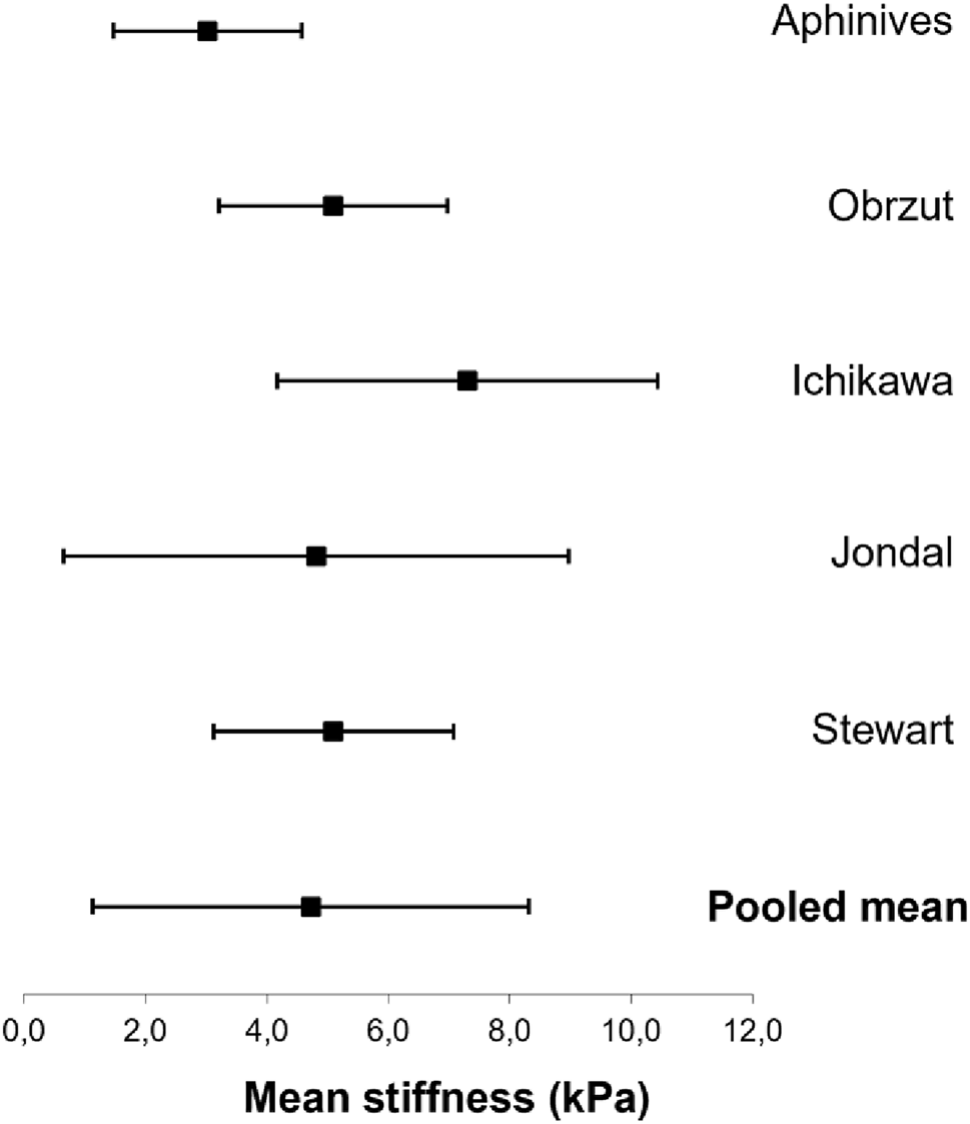
Mean stiffness (kPa) per study and pooled results in patients with leiomyomas

Three of the five studies (Jondal et al.* n* = 102, Ichikawa et al.* n * = 11, and Aphinives et al.* n* = 26) reported that most of the patients, ranging from 62 to 100% had multiple leiomyomas [32, 33, 36]. Among the studies reporting on leiomyoma size (Stewart et al. * n* = 6, Jondal et al. * n* = 102 and Obrzut et al. * n* = 12), the diameter ranged from 4 cm to 22.5 cm [31, 32, 34]. The mean stiffness of leiomyomas ranged from 3.02 to 7.10 kPa across all the included studies. The pooled mean with pooled SD was 4.72 ± 1.83 (Fig.2). Other features are listed in Table 4**.**

**Table 4:** MRI and MRE Features of Leiomyomas

Author	Leiomyoma volume (cm^3^)	Leiomyoma diameter (cm)	Number of leiomyomas	Location of leiomyoma in uterus wall	Leiomyoma characteristics on T2-WI	Leiomyoma characteristics on post-contrast	Stiffness on MRE Mean ± SD /median, range (kPa)
Stewart, [31]	N.R	Mean: 13.86*^∅^ Range 4.5–22.5^∅^	N.R	N.R	Dark: 4 Heterogenous: 1 N.A.: 1	Heterogenous: 1 Homogenous: 3 No enhancement: 1 N.A.: 1	Mean 5.09 ± 1.01* Range 3.95–6.68
Jondal, [32]	Mean: 283.0 ± 398.0	Range 4.5—22.5	Single: 21 Multiple: 81	Submucosal: 17 Intramural: 75 Subserosal: 6 Pedunculated: 4	Dark min. heterogeneity: 69 Dark substantial heterogeneity: 20 Iso/Hyperintense: 13	Greater/equal^: 59 Less^: 31 None^:7 No Gd administered: 5	Mean 4.81 ± 2.12
Ichikawa^#^, [33]	Mean: 412.1 Range 29.3 −864.6)	N.R	Single:0 Multiple:11 (Mean 8 Range 2–17)	Submucosal: 5 Intramural: 12	SI ratio of leiomyoma-to-muscle: mean 1.23, range (0.81–1.84)	N.R	Mean 7.3 ± 1.60* Range 5.2–10.3
Obrzut, [34]	N.R	Median: 6.851^∅^ Range 4 −10.9^∅^	N.R	N.R	N.R	N.R	Mean 5.09 ± 0.96* Median 4.9 Range 3.7–6.9
Aphinives, [35]	Mean: 237.74 ± 187.63 Range 56.24—716.35^#^	N.R	Single: 10 Multiple: 16	N.R	N.R	N.R	Mean 3.02 ± 0.79 Range 1.83–5.06

*N.R.* Not recorded, *SI* Signal intensity, * mean and SD calculated using available data, ^ enhancement compared to myometrium, ^∅^ measurement corresponds to largest leiomyoma, ^#^ in case of multiple leiomyomas, the total was summation of all leiomyomas

#### Association between leiomyoma stiffness and T2 weighted signal intensity or contrast enhancement

In the study conducted by Stewart et al., 50% of the patients (3 out of 6) demonstrated homogenously hypointense leiomyomas on T2-WI, which corresponded with higher mean stiffness values. Conversely, one patient with a heterogenous leiomyoma on T2-WI showed the lowest mean stiffness [31]. 

Similarly, Jondal et al. (*n* = 102) found a correlation between T2 signal characteristics and stiffness values. Their study reported that hyperintense leiomyomas on T2-WI had significant lower stiffness than hypointense, minimally heterogenous leiomyomas. The mean stiffness difference between the two groups was 2.38 kPa: hyperintense leiomyomas had a mean stiffness of 2.88 ± 0.98 kPa, whereas hypointense leiomyomas measured 5.27 ± 2.16 kPa (p = 0.0147). Notably, mean stiffness did not differ significantly across varying contrast enhancement patterns [32].

#### Association between leiomyoma stiffness and histological composition

Obrzut et al. (*n* = 12) examined the relationship between mean stiffness and histological composition. Surgical specimens were stained to differentiate muscle fibers, collagen fibers, and nuclei, and were categorized based on the percentage of connective tissue content. Leiomyomas containing more than 30% fibrous tissue exhibited a higher median stiffness (6.15 kPa) compared to those with up to 15% fibrous content (4.46 kPa) and between 15 and 30% (5.78 kPa) [34].

#### Association between leiomyoma stiffness and treatment outcome

Ichikawa et al. (*n *=11) compared treatment stiffness values in patients undergoing MR-guided focused ultrasound and found that patients who experienced a substantial volume reduction had significantly higher pre-treatment stiffness values (mean 8.3 kPa, range 6.9–10.3) compared to patients without substantial volume reduction (mean 6.1 kPa, range 5.2–8.0) [33].

### Adenomyosis

In one study MRE was evaluated in four patients with suspected adenomyosis (diffuse* n* = 3; focal  *n*  = 1) and one healthy volunteer (Jain 2025) [36]. Two patients underwent hysterectomy, and histologic analysis of the tissue samples was performed. Uterine stiffness was higher in women with adenomyosis (2.93 kPa; range, 2.34–3.39 kPa) than in the healthy volunteer (2.04 kPa).

### Methodological quality assessment

All included studies were either pilot or feasibility studies and were rated as having good methodological quality according to the National Institutes of Health (NIH) quality Assessment Tool for Case Series Studies. Table 5**.**

**Table 5:** Methodological quality assessment of included articles

Author	Q1	Q2	Q3	Q4	Q5	Q6	Q7	Q8	Overall quality
Stewart, [31]	YES	YES	NR	NO	YES	YES	YES	YES	Good
Jondal, [32]	YES	YES	NR	YES	YES	YES	YES	YES	Good
Ichikawa, [33]	YES	NO	YES	YES	YES	YES	YES	YES	Good
Obrzut, [34]	YES	YES	NR	YES	YES	YES	YES	YES	Good
Aphinives, [35]	YES	NO	NR	YES	YES	YES	YES	YES	Good
Juan, [36]	YES	NO	NR	YES*	YES	YES	YES	YES	Good
Q1. Was the study question or objective clearly stated? **Yes, if clearly described in the introduction**									
Q2. Was the study population clearly and fully described? **Yes, if inclusion and exclusion criteria, patient age, and menstrual status are clearly stated**									
Q3. Were the cases consecutive? **Yes, if clearly mentioned in the methods**									
Q4. Were the subjects comparable? **Yes, if only patients with either leiyomomas, deep endometriosis or adenomyosis were included**									
Q5. Was the intervention clearly described? **Yes, provided that MRE techniques are clearly defined**									
Q6. Were the outcome measures clearly defined, valid, reliable, and implemented consistently across all study participants? **Yes, if MRE evaluation was done in all patients in the same way to calculate stiffness**									
Q7. Were the statistical methods well-described? **Yes, if it is reproducible and all details are mentioned**									
Q8. Were the results well-described? **Yes, if all results matched the method section**									
**Quality Rating.** If 6–8 questions were answered with YES, the quality rating is assessed as “Good”. If 4–5 questions were answered with YES, the quality was assessed as ‘Fair’’. All other cases were assessed as poor quality									

NR: Not reported; *Feasibility study including a healthy volunteer

## Discussion

This systematic review identified four pilot studies and two feasibility studies investigating the use of MRE for evaluating fibrosis in benign uterine disorders, specifically leiomyomas and adenomyosis [31–36]. Despite their small scale, findings suggest that MRE is a technically feasible imaging modality to assess fibrosis.

*MRE in leiomyomas- *For leiomyomas, the studies suggest that MRE-derived stiffness could serve as a biomarker of fibrosis. Jondal et al. [32]and Stewart et al. [31] found that T2-hypointense leiomyomas showed higher stiffness values, consistent with Oguchi et al. [37], who linked low T2 signal with reduced proliferative activity and increased fibrosis. Obrzut et al. [34] confirmed this relationship histologically, showing significantly higher stiffness in leiomyomas with > 30% fibrous content (6.15 kPa) compared to less fibrotic lesions (4.46 kPa). Aphinives et al. [35]. also reported leiomyoma stiffness exceeding that of normal myometrium, while Stewart et al. [31] noted values comparable to fibrotic liver disease.

MRE may also help predict treatment outcomes. Ichikawa et al. [33] suggested that leiomyomas with high stiffness respond better to MR-guided focused ultrasound, while those with high T2 signal and high-water content may resist ablation. This likely reflects treatment mechanisms: thermal ablation is more effective in dense fibrotic tissue, whereas embolization depends on vascular supply and may be less effective in poorly perfused leiomyomas. Consistent with this, Chung et al. [38] reported that leiomyomas with high T2 signal intensity were more likely to respond favorably to uterine artery embolization (UAE).

*MRE in adenomyosis- *For adenomyosis, evidence is more limited. In Jain et al. [36], regions of increased stiffness on MRE corresponded with adenomyotic areas on MRI and with histological fibrosis in hysterectomy specimens, strengthening the hypothesis that MRE reflects disease-related remodeling. While preliminary, these results indicate that MRE may provide a non-invasive means to detect and quantify fibrosis in adenomyosis, with potential implications for diagnosis and monitoring.

A major strength of this review is the inclusion of studies that employed quantitative stiffness measurements, providing objective data on uterine tissue properties. Across the available evidence, a consistent MRE set-up was used, with all studies applying the same driver system (a passive driver over the lower abdomen and an active driver producing 60 Hz vibrations), which supports technical comparability. To our knowledge, this is the first systematic review to specifically evaluate MRE for fibrosis in benign uterine disorders, namely leiomyomas and adenomyosis.

However, this systematic review highlights several important limitations in the current evidence base on MRE for benign uterine disorders. A key constraint across the included studies is the absence of large cohort data, which significantly limits the generalizability of the findings. Moreover, the small number of studies, heterogeneity in uterine pathologies investigated (five on leiomyomas and one on adenomyosis), and variability in outcome measures precluded the performance of a meta-analysis.

Also, the studies fail to elaborate on the criteria for patient selection, which may have introduced selection bias. The reported mean age ranged from 40.5 to 49 years, and among the 120 patients for whom reproductive status was reported, 24.2% were perimenopausal and 5.8% were post-menopausal. This is noteworthy, as leiomyomas are generally most clinically significant in women of reproductive age, particularly in those with a desire to conceive.

Technical variability further complicates interpretation. The studies applied different MRI acquisition sequences; 2D gradient-recalled echo (GRE) and spin echo–echo planar imaging (SE-EPI)—which have been reported to differ in technical reliability at various field strengths. Kim et al. [39] showed that GRE had higher failure rates at 3 T compared to 1.5 T, while SE-EPI performed more reliably at 3 T. Reflecting this, Resoundant, a Mayo Clinic–founded company supporting MRE technology, recommends GRE at 1.5 T and SE-EPI at 3 T; most studies in this review followed these guidelines, suggesting appropriate sequence selection despite heterogeneity.

Although all studies used the same MRE system, details of the inversion algorithms used to calculate stiffness maps were not reported, limiting comparability, as different algorithms can affect stiffness measurements.

Only three of the six studies specified who placed ROIs, however none of the studies reported inter or intra-observer variability, and reproducibility seems to be major limitation in imaging studies; different way of ROI placements can also affect stiffness measurements.

Furthermore, none of the studies included a reference ROI in the normal myometrium, so normal values are missing.

Scan times were generally short (< 1 min in most studies), suggesting feasible integration into MRI workflows. However, variability remains (e.g., Jain et al.) and the additional time needed for post-processing and interpretation is not well quantified, highlighting the need for workflow optimization [36].

Limitations of this review itself include the small pool of eligible studies and the inability to perform a meta-analysis. These reflect the early stage of research in this field rather than shortcomings of the review methodology.

## Conclusion

Despite the limited number of available studies, current findings suggest that MRE is a feasible imaging modality for measuring fibrosis in benign uterine disorders.

## Clinical relevance

However, at this stage, these data cannot be translated to clinical practice, due to small number of studies performed (pilot and feasibility studies), missing data on normal stiffness values, technical heterogeneity (different algorithms), different interpretation aspects (ROI placement variability) of uterine stiffness by MRE and missing data on time and costs.

## Recommendations

Future research should aim to move the technique beyond experimental use toward clinical applicability (e.g. for differentiation between symptomatic and asymptomatic leiomyomas, and for guiding the choice between surgical, embolization, and ablative therapies). 

Prospective studies should be designed:

1) including all patients undergoing MRI in routine clinical practice, rather than focusing solely on those scheduled for surgery, to ensure representative and generalizable findings. In this case, the time and accompanying costs of the additional MRE could be limited.

2) with standardization of MRE acquisition protocols, as different algorithms will have effect on measurements and with standardization of fibrosis assessment methods, as different way of ROI placements will also have effect on measurements.

3) with incorporation of normal myometrial measurements as reference values (e.g. as additional sequence in patient undergoing pelvic MRI for other conditions) and also reporting other factors such as tissue cellularity, vascularity, edema, hormonal status, as intrinsic heterogeneity of uterus masses will also have effect on quantifying tissue properties (fibrosis).

4) to validate MRE-derived stiffness measurements against histopathological results or to assess its added value alongside conventional imaging modalities, particularly in relation to disease progression, symptom severity, treatment planning, and patient-reported outcomes.

Such studies may help establish the potential of MRE as a clinically valuable tool for assessing fibrosis in benign uterine disease.

## Supplementary Information

Below is the link to the electronic supplementary material.

Supplementary file1 (DOCX 572 KB)

## Data Availability

All extracted data are available in tables and supplementary files. Details on the search results is available on request (endnote files).
