# Supplementary material for: Magnetic resonance elastography (MRE) for the evaluation of fibrosis in patients with benign uterine disorders: a systematic review
Source: Abdom Radiol (NY). 2026 Feb 5;51(8):4106–17. doi: 10.1007/s00261-026-05374-8 (PMC13388771; doi:10.1007/s00261-026-05374-8)
Supplement: Supplementary file 1 — Supplementary file1 (DOCX 572 KB) [file 261_2026_5374_MOESM1_ESM.docx]

**Supplement A. *Search strategy***

PUBMED

| #1 | (("magnetic resonance imaging"[MeSH Terms] OR ("magnetic"[All Fields] AND "resonance"[All Fields] AND "imaging"[All Fields]) OR "magnetic resonance imaging"[All Fields])) |
| --- | --- |
| #2 | "elasticity imaging techniques"[MeSH Terms] OR ("elasticity"[All Fields] AND "imaging"[All Fields] AND "techniques"[All Fields]) OR "elasticity imaging techniques"[All Fields] OR "elastographies"[All Fields] OR "elastography"[All Fields] |
| #3 | #1 AND #2 |
| #4 | "fibroids"[All Fields] OR "leiomyoma"[MeSH Terms] OR "leiomyoma"[All Fields] OR "fibroid"[All Fields] OR "fibroids"[All Fields] |
| #5 | "leiomyoma"[MeSH Terms] OR "leiomyoma"[All Fields] OR "leiomyomas"[All Fields] |
| #6 | ("endometriosis"[MeSH Terms] OR "endometriosis"[All Fields] OR "endometrioses"[All Fields]) AND ("endometriosis"[MeSH Terms] OR "endometriosis"[All Fields] OR "endometrioses"[All Fields]) |
| #7 | "adenomyosis"[MeSH Terms] OR "adenomyosis"[All Fields] OR "adenomyoses"[All Fields] |
| #8 | #4 OR #5 OR #6 OR #7 |
| #9 | # 3 AND #8 |

EMBASE

| #1 | 'nuclear magnetic resonance imaging'/exp OR 'nuclear magnetic resonance imaging' |
| --- | --- |
| #2 | "'elastography'/exp OR 'elastography' |
| #3 | #1 AND #2 |
| #4 | 'fibroids'/exp OR ‘fibroids’ |
| #5 | 'leiomyoma'/exp OR ‘leiomyoma’ |
| #6 | 'endometriosis'/exp OR 'endometriosis' |
| #7 | 'adenomyosis'/exp OR 'adenomyosis' |
| #8 | #4 OR #5 OR #6 OR #7 |
| #9 | # 3 AND #8 |

*exp= broad search, all field included

**Supplement B. *Calculation of pooled mean and pooled SD***

**Pooled mean was calculated as follows:**

$$\frac{\left( \mu1*n1 \right)+\left( \mu2*n2 \right)+\left( \mu3*n3 \right)+\left( \mu4*n4 \right)+\left( \mu5*n5 \right)}{\left( n1+n2+n3+n4+n5 \right)}$$

**Pooled SD (standard deviation) was calculated as follows:**

$$\sqrt{\begin{aligned} \text{ }\text{ (}n1\text{-1)S}\text{1}\text{2 }\text{+ (}n2\text{-1)S}\text{2}\text{2 }\text{+ (}n3\text{-1)S}\text{3}\text{2 }\text{+}\text{ }\text{(}n4\text{-1)S}\text{4}\text{2 }\text{+ (}n5\text{-1)S}\text{5}\text{2}\text{ } \\ \text{ } \\ (n1+n2+n3+n4+n5) \text{- k}\text{ }\text{ } \end{aligned}}$$

$\mu=mean, n=number of patients$*,* $\text{k}$ *=total number of studies*

**Supplement C. *National Institutes of Health (NIH): Quality Assessment Tool***

| **The National Institutes of Health (NIH) quality assessment tool for case-series study**  **https://www.nhlbi.nih.gov/health-topics/study-quality-assessment-tools** | | | |
| --- | --- | --- | --- |
| **Major Components** | **Response options** | | |
| 1. Was the study question or objective clearly stated? **Yes, if clearly described in the introduction.** | Yes | No | Other (CD, NA, NR) |
| 2. Was the study population clearly and fully described? **Yes, if inclusion and exclusion criteria, patient age, and menstrual status are clearly stated.** | Yes | No | Other (CD, NA, NR) |
| 3. Were the cases consecutive? **Yes, if clearly mentioned in the methods.** | Yes | No | Other (CD, NA, NR) |
| 4. Were the subjects comparable? **Yes, if only patients with either leiyomomas, deep endometriosis or adenomyosis were included.** | Yes | No | Other (CD, NA, NR) |
| 5. Was the intervention clearly described? **Yes, provided that MRE techniques are clearly defined.** | Yes | No | Other (CD, NA, NR) |
| 6. Were the outcome measures clearly defined, valid, reliable, and implemented consistently across all study participants? **Yes, if MRE evaluation was done in all patients in the same way to calculate stiffness.** | Yes | No | Other (CD, NA, NR) |
| 7. Were the statistical methods well-described? **Yes, if it is reproducible and all details are mentioned.** | Yes | No | Other (CD, NA, NR) |
| 8. Were the results well-described? **Yes, if all results matched the method section.** | Yes | No | Other (CD, NA, NR) |
| **Quality Rating** If 6-8 questions were answered with YES, the quality rating is assessed as “Good”. If 4-5 questions were answered with YES, the quality was assessed as ‘Fair’’. All other cases were assessed as poor quality | Good | Fair | Poor |

***^#^*** *Multi-center indicating involvement of authors from different institutions; ^@^ studies performed at the same institute*

**Supplement D. *MRI Protocol Characteristics***

| **Author**  **(year of publication)** | **MRI Vendor** | **MRI Field Strength** | **Coil Type** | **Sequences in MRI protocol** |
| --- | --- | --- | --- | --- |
| Stewart,  2011 | Signa, GE Healthcare, Milwaukee, WI, USA | 1.5T | Four-element phased-array coil | T2 axial or sagittal  T1 |
| Jondal,  2018 | Signa, GE Healthcare, Milwaukee, WI, USA | 1.5T | Phased-array pelvic coil | T2 axial, sagittal and coronal  T1 axial, sagittal and coronal pre/post-Gd* |
| Ichikawa,  2019 | Signa Excite, version 12; GE Medical Systems, Waukesha, WI, USA | 1.5T | 8 channel phased- array coil | T2 axial, coronal, sagittal  T1 fat sat post contrast axial, coronal, sagittal |
| Obrzut,  2020 | Optima, GE Healthcare, Milwaukee, WI, USA | 1.5T | 16 channel phased-array coil | Not specified, but at least T2 |
| Aphinives,  2023 | Achieva dStream, version 5.6.1.0, Philips Healthcare | 3.0T | Phased-array surface coil | T2 axial |
| Juan,  2025 | Skyra Fit, Siemens Healthineers, Erlangen, Germany | 3.0T | 32-channel spine coil  30-channel body matrix coil | T2 axial |

**Gd: (Intravenous) Gadolinium*

**Supplement E. *MRE Technical Parameters***

| **Author**  **(year of publication)** | **MRI sequence** | **Repetition Time**  **(ms)** | **Echo Time**  **(ms)** | **Slice thickness**  **(mm)** | **Field of View (FOV)**  **(cm)** | **Matrix size** | **Flip angle**  **(°)** | **Imaging planes** | **Number of slices** | **Scan time** |
| --- | --- | --- | --- | --- | --- | --- | --- | --- | --- | --- |
| Stewart,  2011 | 2D GRE-based | 50 | 24,9 - 28,5 | 4-5 | 24-42 | 256 x 64 | 30 | Axial or sagittal (depending on fibroid location) | N.R. | N.R. |
| Jondal,  2018 | 2D GRE-based | 50 | 18,4 | 10 | 24-42 | 256 x 64 | 30 | Axial | 4 | 59 sec (4 breath -holds) |
| Jondal,  2018 | 3D SE-SPI^*^ | 1333,8* | 44,0* | 3,5* | 44,8* | 96 x 96* | N.R. | Axial* | 32* | 64 sec (4 breath holds)* |
| Ichikawa,  2019 | SE-EPI | 500 | 50 | 5 | 40 x 40 | 96 x 96 | 30 | Axial | 7 | 38 sec (2 acquisitions) |
| Obrzut,  2020 | 2D GRE-based | 33 | 20 | 10 | N.R. | 256 x 256 | 30 | N.R. | N.R. | N.R. |
| Aphinives,  2023 | 2D Phase Contrast FFE | N.R. | N.R. | N.R. | N.R. | N.R. | N.R. | Axial | N.R. | N.R. |
| Jain,  2025 | 3D EPI | 6,400 | 79 | 3 | 24 | 80 x 80 | N.R. | Axial | N.R. | 5 min 33 sec |

**subset of patients underwent additional 3D MRE, GRE: gradient-recalled echo-based pulse sequence, SE-EPI: spin echo-echo planar imaging, FFE: Fast Field echo*
